# Resilience, Quality of Life, and Minor Mental Disorders in Nursing Professionals: A Study in Challenging Work Environments

**DOI:** 10.3390/ijerph22091375

**Published:** 2025-08-31

**Authors:** Emerson Roberto dos Santos, Marco Antonio Ribeiro Filho, Weslley dos Santos Borges, William Donegá Martinez, João Daniel de Souza Menezes, Matheus Querino da Silva, André Bavaresco Gonçalves Cristóvão, Renato Mendonça Ribeiro, Flávia Cristina Custódio, Geovanna Mohieddine Felix Pereira, Jéssica Gisleine de Oliveira, Alex Bertolazzo Quitério, Rauer Ferreira Franco, Amanda Oliva Spaziani, Ana Paula Bernardes da Rosa, Rodrigo Soares Ribeiro, Nayara Tedeschi Fernandes Furtile, Daniele Nunes Longhi Aleixo, Tânia Cassiano Garcia Gonçalves, João Júnior Gomes, Adriana Pelegrini dos Santos Pereira, Fernando Nestor Facio Júnior, Marli de Carvalho Jerico, Josimerci Ittavo Lamana Faria, Maysa Alahmar Bianchin, Luís Cesar Fava Spessoto, Maria Helena Pinto, Rita de Cássia Helú Mendonça Ribeiro, Daniele Alcalá Pompeo, Antônio Hélio Oliani, Denise Cristina Móz Vaz Oliani, Júlio César André, Daniela Comelis Bertolin

**Affiliations:** 1 Center for Studies and Development of Health Education (CEDES), Faculty of Medicine of São José do Rio Preto (FAMERP), Avenida Brigadeiro Faria Lima, 5416, São José do Rio Preto 15090-000, São Paulo, Brazil; emerson.santos@edu.famerp.br (E.R.d.S.); marcoribeirofilho@gmail.com (M.A.R.F.); william.martinez@edu.famerp.br (W.D.M.); fccustodio@funecsantafe.edu.br (F.C.C.); jessica.gisleine@edu.famerp.br (J.G.d.O.); alex.quiterio@edu.famerp.br (A.B.Q.); daniele.aleixo@edu.famerp.br (D.N.L.A.); fernando.nestor@famerp.br (F.N.F.J.); marli@famerp.br (M.d.C.J.); josimerci.faria@edu.famerp.br (J.I.L.F.); maysa@famerp.br (M.A.B.); julio.andre@edu.famerp.br (J.C.A.); 2 Faculty of Medicine, University of Brazil, Fernandópolis 15600-000, São Paulo, Brazil; weslley.borges@ub.edu.br; 3 Faculty of Medicine of São José do Rio Preto (FAMERP), São José do Rio Preto 15090-000, São Paulo, Brazil; matheus.querino@edu.famerp.br (M.Q.d.S.); nayarafnd@yahoo.com.br (N.T.F.F.); taniagarcia_jb@hotmail.com (T.C.G.G.); jjenf@terra.com.br (J.J.G.); adrianapelegrini@famerp.br (A.P.d.S.P.); cspessoto@gmail.com (L.C.F.S.); mariahelena@famerp.br (M.H.P.); ritadecassia@famerp.br (R.d.C.H.M.R.); daniele.pompeo@famerp.br (D.A.P.); 4 University of Santo Amaro (UNISA), São Paulo 04743-030, São Paulo, Brazil; andre.cristovao017@gmail.com; 5 Ribeirão Preto School of Nursing, USP, Ribeirão Preto 14040-902, São Paulo, Brazil; drrenatoribeiroenf@gmail.com; 6 University Nove de Julho, São Paulo 01504-001, São Paulo, Brazil; geovannamohieddine@gmail.com; 7 University of Brazil, Fernandópolis 15600-000, São Paulo, Brazil; rauerf@hotmail.com (R.F.F.); spazianimedicina@gmail.com (A.O.S.); 8 Centro Universitário do Norte de São Paulo (UNORTE), São Jose do Rio Preto 15020-040, São Paulo, Brazil; paulabernardes@unorte.edu.br; 9 University Hospital Center Cova da Beira, University of Beira Interior, 6200-251 Covilhã, Portugal; oliani@famerp.br (A.H.O.); vaz.oliani@gmail.com (D.C.M.V.O.); 10 União das Faculdades os Grandes Lagos (UNILAGO), São José do Rio Preto 15030-070, São Paulo, Brazil; danielacomelisbertolin@gmail.com

**Keywords:** mental health, minor mental disorders, resilience, quality of life, nursing work environment

## Abstract

Introduction: The mental health of nursing professionals is an escalating global concern, particularly due to the inherently challenging work conditions they frequently encounter. This study aimed to investigate the prevalence of Minor Mental Disorders (MMD) and resilience levels among nursing professionals, analyzing the relationship between these constructs and identifying resilience’s potential protective role. Methods: This was a quantitative, descriptive, correlational, and cross-sectional study. The sample consisted of 203 nursing professionals (including nursing assistants, technicians, and nurses) from two healthcare institutions in the interior of São Paulo, Brazil. Data were collected between August and October 2019. Instruments utilized included a sociodemographic and professional questionnaire, the Self-Report Questionnaire (SRQ-20) for MMD screening, and the Wagnild & Young Resilience Scale. Results: The overall prevalence of MMD in the studied sample was 31.0%. Mean scores for the SRQ-20 domains were observed as follows: Depressive/Anxious Mood (1.33), Somatic Symptoms (1.63), Reduced Vital Energy (1.77), and Depressive Thoughts (0.39). A key finding indicated that resilience did not demonstrate a significant direct predictive role on MMDs when the effect of quality of life was controlled. However, resilience showed a significant positive correlation with Quality of Life (QoL) (coef. = 0.515; *p* < 0.001). Furthermore, QoL emerged as a robust and statistically significant negative association with all dimensions of MMD. Discussion: These findings suggest that resilience may function as an indirect moderator or precursor to QoL, with QoL, in turn, exerting a more direct and substantial influence on the reduction of MMDs. This integrated perspective aligns with the understanding that resilience contributes to a more adaptive assessment of stressors and, consequently, to better QoL, thereby minimizing the detrimental effects of stress on mental health. Conclusion: This study reaffirms the high prevalence of Minor Mental Disorders among nursing professionals, highlighting Quality of Life as a primary target for interventions aimed at promoting mental well-being. It also emphasizes resilience as a valuable individual resource that indirectly supports mental health by enhancing QoL. A holistic understanding of occupational stressors, psychosocial, and biological mechanisms is crucial for developing effective and targeted support strategies for these essential professionals.

## 1. Introduction

The mental health of nursing professionals has emerged as a growing global concern, especially when considering the challenging working conditions to which these professionals are frequently exposed. In a worldwide scenario where the demand for healthcare services is constant and, at times, overwhelming, physical and emotional overload has become an intrinsic reality in these workers’ routines. Nursing practice is commonly performed under conditions that may be inadequate, with hierarchical relationships that are sometimes conflictual, factors that significantly contribute to occupational stress and, consequently, to physical and emotional exhaustion [1,2,3,4]. Specifically in Brazil, nursing professionals often operate within a public healthcare system (Sistema Único de Saúde—SUS) characterized by high patient volumes, limited resources, and intense pressure to deliver care efficiently despite systemic constraints [5]. These particularities of the Brazilian work environment exacerbate the existing global challenges, leading to unique stressors that impact the mental well-being of nurses [6]. The physiological response to these stressors can be effectively understood through Hans Selye’s General Adaptation Syndrome (GAS), a model positing that chronic exposure to extreme demands may lead to the exhaustion phase, thereby undermining the organism’s sustainable coping capacity [7]. These factors, combined with excessive working hours and constant pressure, result in high rates of absenteeism and deterioration in the quality of life (QoL) of these individuals [8,9,10].

Engagement with patients in critical situations, in inpatient units or emergency departments, exposes nursing professionals to a particularly intense level of physical and psychological stress. According to the Job Demands-Resources (JD-R) Model, nursing is characterized by high job demands—such as the ‘requirement for significant physical exertion,’ the need to ‘stand for long periods,’ and a ‘fast-paced work rhythm’—which, in the absence of adequate resources, can lead to exhaustion [11]. Emergency departments, for example, are described as physically and psychologically overwhelming environments, making nursing staff more susceptible to emotional exhaustion and at risk of developing traumatic stress [12]. This exhaustion constitutes a core component of burnout, which further encompasses depersonalization and diminished personal accomplishment [13]. These symptoms collectively underscore the challenge of sustaining engagement and high-quality care within an environment of persistent pressure. The requirement for significant physical effort, such as standing for long periods, repetitive use of hands, and manual labor, combined with an accelerated work pace and time pressure, contributes to a high prevalence of musculoskeletal pain and other physical problems [14,15,16]. Furthermore, coping with pain, suffering, and death, team anxiety, and rigid work routines add complex layers to daily stress, directly impacting the quality of life and mental well-being of these professionals [17].

In this context, mental health and quality of life are conceptualized through the lens of the Biopsychosocial Model, which posits health and illness as outcomes of a complex interplay among biological, psychological, and social factors [18]. This implies that nurses’ working conditions and personal experiences not only influence their psychological state but can also manifest profound biological correlates. The Quality of Life (QoL), defined as the individual’s subjective perception of their position in life, considering the cultural and value context, their goals, expectations, standards, and concerns, is a crucial element [19]. Although measuring QoL is complex, instruments such as WHOQOL-BREF, a widely validated abbreviated version of WHOQOL-100, allow for the assessment of the impact of various conditions on an individual’s life, covering physical, psychological, social relationships, and environmental domains [20,21]. Recent studies, including the research that underpins this manuscript, have revealed that nursing professionals frequently exhibit levels of quality of life that indicate dissatisfaction, which is a direct reflection of the demands of their profession [22,23].

Dissatisfaction with the quality of life and exposure to chronic stressors contribute to the high prevalence of Minor Mental Disorders (MMD) among nursing professionals. MMDs are clinically significant conditions that manifest through alterations in mood, emotions, thinking, and behavior, frequently associated with personal distress or impaired functioning [24,25]. Symptoms such as forgetfulness, fatigue, depression, lack of concentration, irritability, and insomnia are common among these professionals [26]. The nursing staff is, in fact, considered a risk group for MMD-related illness, with nursing assistants, technicians, and nurses ranking among the positions with the highest prevalence and risk of work leave due to these conditions [27,28,29]. The Self-Report Questionnaire (SRQ-20), developed by the World Health Organization (WHO), is an instrument widely used in Brazil to screen for these disorders, demonstrating its relevance in the early identification of MMDs in the professional population [30,31].

In contrast to risk factors and minor mental disorders, resilience (R) emerges as an important protective and adaptive factor. Derived from the Latin “resilio,” resilience is defined as the capacity of an individual or group to positively construct or reconstruct themselves, even in the face of adversities and in unfavorable environments [32,33]. In the nursing work context, resilience is a continuous process of personal growth and development of potentialities that allow professionals to cope with the exhausting work pace, pressure, and responsibilities, minimizing stressors and promoting well-being [34,35,36]. The capacity for resilience, assessed by scales such as Wagnild & Young’s, is fundamental for these professionals to maintain their mental health and quality of life in such a demanding environment [37]. Additionally, positive psychology emphasizes resilience as a human strength that enables individuals to thrive in the face of challenges, contributing to well-being and the prevention of psychopathology [38].

The complexity of these previously discussed interactions, encompassing the relationship between occupational stressors, quality of life, minor mental disorders, and resilience, becomes even more apparent when considering the underlying biological and psychophysiological mechanisms. Chronic occupational stress exposure can lead to dysregulation of the Hypothalamic–Pituitary–Adrenal (HPA) axis, resulting in altered cortisol levels and impacting the Autonomic Nervous System (ANS), with sympathetic nervous system predominance and a consequent reduction in heart rate variability. These biological alterations directly contribute to the symptoms of anxiety, depression, and fatigue observed in Minor Mental Disorders (MMDs). Furthermore, chronic stress can affect neurotransmitter systems (such as serotonin and dopamine) and neural plasticity in brain regions critical for emotional and cognitive regulation, including the prefrontal cortex, amygdala, and hippocampus [39]. There is also growing evidence that prolonged stress can induce neuroinflammation and oxidative stress, processes that contribute to the pathogenesis of mental disorders and the impairment of quality of life [40]. Resilience, in turn, is associated with more effective regulation of these biological systems, allowing for a more rapid recovery of homeostasis following stressful events and conferring protection against the deleterious effects of chronic stress.

Given the complexity and the scarce scientific production focused on the relationship between quality of life, minor mental disorders, and resilience, especially in nursing literature, the relevance of this theme is undeniable. Most studies focused on the mental health of nursing professionals tend to concentrate on isolated aspects of suffering or stress, without deepening the interconnection between these constructs and the role of resilience as a crucial protective factor [41]. Existing research often examines these variables in isolation or in simple bivariate relationships (e.g., resilience and mental health, or quality of life and mental health) [42,43]. However, there is a significant gap in studies that integrate resilience and Quality of Life as interacting variables within a comprehensive model to predict MMD. Specifically, previous research has not adequately explored the potential indirect or mediating role of Quality of Life in the relationship between resilience and the development of MMDs. Understanding this dynamic, by integrating psychosocial perspectives and biological mechanisms, is fundamental for developing more effective and targeted interventions, both at the individual and organizational levels, aimed at promoting mental health and preventing illness.

**Figure 1:**
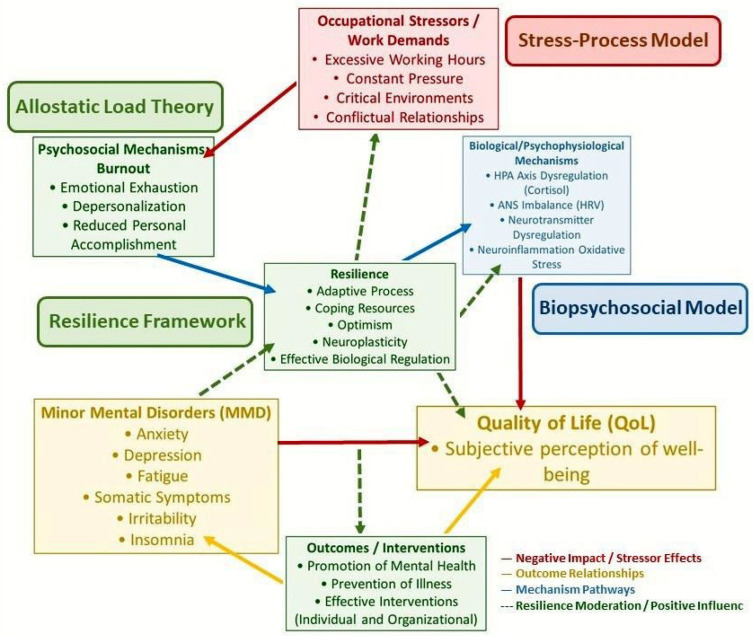
Integrated theoretical model: psychological health and well-being of nursing professionals.

The intricate interplay among occupational stressors, psychosocial and biological mechanisms, Minor Mental Disorders (MMD), Quality of Life (QoL), and the protective role of Resilience, specifically within the context of healthcare professionals, particularly nursing, is illustrated in Figure 1. This integrated theoretical model hypothesizes specific directional relationships: (a) occupational stressors contribute to MMD through both psychosocial and biological mechanisms; (b) higher Quality of Life directly predicts a reduction in MMD symptoms; and (c) resilience positively influences Quality of Life, thereby indirectly protecting against MMD. This conceptualization positions Quality of Life as a crucial mediator between resilience and mental health outcomes.

Based on this theoretical framework and the identified research gaps, this study proposes the following hypotheses:

- H1: Higher levels of Quality of Life will be significantly associated with a lower prevalence and severity of Minor Mental Disorders among nursing professionals [44].
- H2: Resilience will be positively correlated with Quality of Life in nursing professionals [45].
- H3: Resilience will indirectly influence the prevalence and severity of Minor Mental Disorders by enhancing Quality of Life among nursing professionals (e.g., resilient individuals experience better QoL, which in turn reduces MMD symptoms) [46].

In this scenario, the present study proposes an in-depth investigation. The main objective of this work is to investigate the prevalence of Minor Mental Disorders (MMDs) and levels of resilience among nursing professionals. It also seeks to analyze the relationship between these two constructs and identify the role of resilience as a potential protective factor, contributing to a more holistic understanding of mental health in nursing and to the development of more effective support strategies.

## 2. Materials and Methods

### 2.1. Study Design

This is a quantitative study with a descriptive, correlational, and cross-sectional design. The quantitative approach was selected to allow for objective measurement of variables and the application of robust statistical analyses, seeking to identify patterns, relationships, and the prevalence of phenomena. The descriptive character aims to characterize the sample and the attributes of the investigated variables, while the correlational aspect proposes to examine the associations between resilience, quality of life, and dimensions of mental health. The choice of a cross-sectional design implies that data were collected at a single point in time, offering a “snapshot” of the relationships between variables at the moment of collection. It is important to emphasize that, due to its cross-sectional nature, this study allows for the identification of associations but cannot establish direct causal relationships or observe changes over time. This study was conducted and reported in accordance with the Strengthening the Reporting of Observational Studies in Epidemiology (STROBE) guidelines (Supplementary File S1).

### 2.2. Sample and Context

The study population consisted of nursing professionals (nursing assistants, technicians, and nurses) from Inpatient Units (IUs) and Emergency Departments (EDs) of the public healthcare network and the Base Hospital (BH) in the municipality of São José do Rio Preto, São Paulo, Brazil. The total estimated population was 444 professionals.

The final study sample consisted of 203 nursing professionals. While recognizing the rigorous requirements for a priori sample size estimation using statistical parameters (e.g., means, standard deviations, and desired effect sizes) to ensure adequate statistical power for hypothesis testing, this descriptive and correlational study adopted a comprehensive approach. The primary objective was to investigate the prevalence and relationships of mental health constructs within the entire accessible population of nursing professionals across the selected healthcare institutions. Consequently, the sample size of 203 participants was obtained through a recruitment process that aimed to include all eligible professionals who provided informed consent during the data collection period, representing approximately 45.7% of the total estimated population within these defined settings. This substantial representation from the target population allows for a detailed characterization of the phenomena under investigation and provides a robust basis for exploring correlations within this specific cohort.

The participating healthcare institutions included the Emergency Care Units (UPAs) of the municipality of São José do Rio Preto and the Base Hospital (BH) of the same city.

The UPAs: The municipal health system of São José do Rio Preto has five UPAs (UPA Jaguaré, UPA Santo Antônio, UPA Tangará/Estoril, UPA Região Norte, UPA Vila Toninho), distributed across different regions of the city (São José do Rio Preto (Municipality), 2019). This study included professionals from all five UPAs.

The Base Hospital (BH): It is one of the largest and most important hospital complexes in the State of São Paulo, being a teaching hospital affiliated with the Faculty of Medicine of São José do Rio Preto (FAMERP). The BH serves most of its patients through the Unified Health System (SUS) and serves as a medical reference center for more than two million inhabitants from 102 neighboring municipalities [47]. It has 708 inpatient beds (including ICUs) and one of the largest emergency services in the interior of São Paulo, with approximately 12,000 monthly consultations, divided into private insurance (EC) and SUS (E-SUS) sectors. From BH, all nine inpatient units and the two emergency departments (EC and E-SUS) were included.

### 2.3. Data Collection

Data collection occurred between August and October 2019. The project was submitted for review by the Research Ethics Committee (REC) of the Faculty of Medicine of São José do Rio Preto (FAMERP), under Certificate of Presentation for Ethical Appraisal (CAAE) n. 89714418.0.0000.5415, and was approved on 2 July 2018, with opinion n. 2,748,173.

Data were collected after prior scheduling with the nursing managers of the participating sectors and institutions. The researchers explained the study objectives to the managers and provided the data collection instruments and the Informed Consent Forms (ICFs). The managers, in turn, passed the materials to the professionals under their management. To ensure participant anonymity, the ICFs (one version for the respondent and another for the researcher) and the completed instruments were collected in separate sealed envelopes and subsequently delivered to the researchers. Professionals on vacation and/or away from activities, as well as those who did not agree to participate, were excluded from the sample.

### 2.4. Instruments

Four self-administered instruments were used for data collection:

- Sociodemographic and Professional Questionnaire: Developed by the researchers, this closed-question instrument collected information on sex, age, marital status, number of children, family income, professional category, work sector and institution, number of hours worked, number of employment relationships, motivation for accumulating jobs, and work shift. For nurses, it also included academic qualifications (specialization, master’s degree, doctorate). As a descriptive data collection tool, this questionnaire does not require psychometric reliability coefficients in the same manner as psychological scales. While Figure 1 illustrates a comprehensive theoretical model including occupational stressors, this questionnaire captured specific contextual and professional characteristics (e.g., workload, number of employment relationships) that serve as relevant indicators within that broader framework, rather than directly measuring constructs like burnout.
- Quality of Life Assessment Instrument (WHOQOL-BREF): Abbreviated version of WHOQOL-100, developed by The WHOQOL Group (1998) and widely validated for use in various cultures [19,20,48]. It consists of 26 questions, the first two about the general quality of life and satisfaction with health, and the remaining 24 distributed across four domains: Physical, Psychological, Social Relationships, and Environment. Responses range from 1 to 5 (from “not at all satisfied” to “very satisfied”). For analysis, raw scores are transformed to a scale of from 0 to 100. Values below 70 are considered unsatisfactory, and equal to or above 70, satisfactory. Validation studies for WHOQOL-BREF have consistently reported good internal consistency, with Cronbach’s alpha values typically ranging from 0.60 to 0.90 for its domains [20,48]. In the current study, the composite reliability was 0.829 for the overall Quality of Life (QoL) factor, 0.561 for Physical, 0.266 for Environmental, and 0.261 for Social. The Psychological factor presented extremely low composite reliability (0.00000983) in our analysis, as detailed in the Results section.
- Self-Report Questionnaire (SRQ-20): Developed by the World Health Organization (WHO) for screening minor mental disorders (MMD), this instrument consists of 20 items with dichotomous responses (yes/no) [49,50]. Each positive response contributes 1 point to the total score (ranging from 0 to 20). The symptoms are grouped into four categories: depressive/anxious mood, somatic symptoms, decreased vital energy, and depressive thoughts [51,52,53]. The Brazilian version of the SRQ-20 has demonstrated validity and reliability in previous studies [30,31,54]. A total score equal to or greater than eight points on the SRQ-20 was used as a criterion for the diagnosis of MMD, as widely adopted in the literature [30]. Original validation studies of the SRQ-20 have reported adequate internal consistency, with Cronbach’s alpha values generally above 0.70 [52,53]. In this study, the composite reliability for the Decreased Vital Energy (DEV) factor was 0.842, for Depressive–Anxious Mood (HuDA) was 0.739, for Depressive Thoughts (PD) was 0.826, and for Somatic Symptoms (SS) was 0.762.
- Wagnild & Young Resilience Scale: This scale, originally developed by Wagnild and Young (1993), assesses the individual’s ability to deal with adversities and maintain well-being [55]. In Brazil, it was translated and adapted, with its validity and reliability confirmed in studies such as Pesce et al. (2005) and Perim et al. (2015) [56,57]. The original scale has 25 items with a 7-point Likert scale (1 = strongly disagree; 7 = strongly agree). Scores range from 25 to 175 points, where higher values indicate greater resilience. In the present study, based on Bayesian confirmatory factor analysis, a 14-item version with collapsed response categories was used, in order to optimize model fit and internal consistency [58,59,60]. A higher score on this scale indicates a higher level of resilience. Validation studies of the Wagnild & Young Resilience Scale have consistently shown high internal consistency, with Cronbach’s alpha values often exceeding 0.90 [55,56]. In the current study, the composite reliability for the 14-item collapsed model was 0.860, indicating high internal consistency.

### 2.5. Data Analysis

Data analysis was divided into three main stages, using advanced statistical approaches to ensure the robustness and accuracy of the results. The analyses were performed using R software 4.4.0 (R Core Team, 2024), with specific packages for statistical modeling [61]. For clarity, key statistical terms and their interpretations are provided, aiming to make the methodology accessible to a broad readership.

#### 2.5.1. Bayesian Confirmatory Factor Analysis (BCFA)

BCFA was employed to investigate the factorial structure of the SRQ-20, Wagnild & Young Resilience Scale, and WHOQOL-BREF instruments. This approach was selected for its flexibility and robustness in handling ordinal data and moderate sample sizes, generating posterior distributions of parameters [62,63,64,65,66,67]. For BCFA, the Markov Chain Monte Carlo (MCMC) algorithm and the blavaan package in R software were used [65]. The fit of Bayesian models was evaluated through the posterior predictive *p*-value (ppp), Deviance Information Criterion (DIC), Watanabe-Akaike Information Criterion (WAIC), and Leave-One-Out Information Criterion (LOOIC) [68]. Good model fit in BCFA is typically indicated by a ppp value close to 0.5, lower values for DIC, WAIC, and LOOIC (relative to alternative models), suggesting better predictive accuracy and parsimony.

For the SRQ-20, a model of four correlated factors (Depressive–Anxious Mood, Somatic Symptoms, Decreased Vital Energy, and Depressive Thoughts) was validated [51,52,53].

For the Resilience Scale, models of 14 and 25 items were tested, with and without collapsed response categories. The 14-item model with collapsed categories demonstrated the best balance of fit.

For the WHOQOL-BREF, Hierarchical, Partial Bifactor, and Complete Bifactor models were evaluated. The Complete Bifactor Model proved to be the most adequate in terms of fit, although the psychological factor presented extremely low composite reliability and was not included in subsequent analyses [69,70,71].

#### 2.5.2. Estimation and Transformation of Factor Scores

After validating the factorial structures, factor scores to represent latent variables were calculated from the 2000 posterior distributions of latent variables generated by BCFA [63,64]. These factor scores serve as quantitative measures of the underlying psychological constructs. To mitigate the problem of factor score indeterminacy and ensure the preservation of internal correlations between factors, whitening and coloring transformations were applied [62,72,73,74,75,76,77,78,79,80]. These transformations standardize the scores while maintaining their relational properties, preparing them for subsequent analyses.

#### 2.5.3. Path Analysis Modeling

Path analysis was used to examine the predictive relationships between WHOQOL-BREF domains and Resilience with dimensions of mental health. This analysis was performed using the lavaan package in R software and was based on the 2000 posterior distributions of transformed factor scores [81]. The quality of fit of the path analysis models was evaluated through indices such as chi-square (χ^2^), degrees of freedom (df), *p*-value, Comparative Fit Index (CFI), Tucker–Lewis Index (TLI), and Root Mean Square Error of Approximation (RMSEA). A good model fit for path analysis is generally indicated by a non-significant chi-square *p*-value (*p* > 0.05), CFI and TLI values greater than 0.90 (preferably > 0.95), and RMSEA values less than 0.08 (preferably < 0.05).

## 3. Results

### 3.1. Sample Characterization

The study sample consisted of 203 nursing professionals. The response rate for this study was 45.7% of the estimated population of 444 professionals, which, while offering valuable insights, should be considered when interpreting the generalizability of findings.

Specifically, of the 203 professionals, 25 (12.3%) were nursing assistants, 99 (48.8%) were nursing technicians, and 79 (38.9%) were nurses. The sample was predominantly female (81.3%) and younger, with 58.6% under 39 years of age. Regarding marital status, 62.1% reported living with a partner, and 61.6% had one or more children. The predominant family income (66.0%) was in the range of BRL 3000 or more per month, which is consistent with middle-income brackets in the region and provides context for the economic status of the participants.

Regarding professional characteristics, 67% of participants worked between 31 and 44 h weekly, and 77% had only one employment relationship. The most prevalent work sector was emergency (71%), with 44% working in Emergency Care Units (UPAs) and 29% in hospital inpatient units. The motivation for accumulating employment relationships, for the 21% who did so, was related to financial necessity (15%) and pursuit of better remuneration (6%). Regarding academic qualifications, 21% of professionals had a specialization, and 3% had a master’s or doctoral degree.

### 3.2. Prevalence of Minor Mental Disorders (MMD)

Screening for Minor Mental Disorders (MMD) was conducted using the Self-Report Questionnaire (SRQ-20). The prevalence of MMD in the studied sample was 31.0% (n = 63), defined by an SRQ-20 score equal to or greater than eight points.

**Figure 2:**
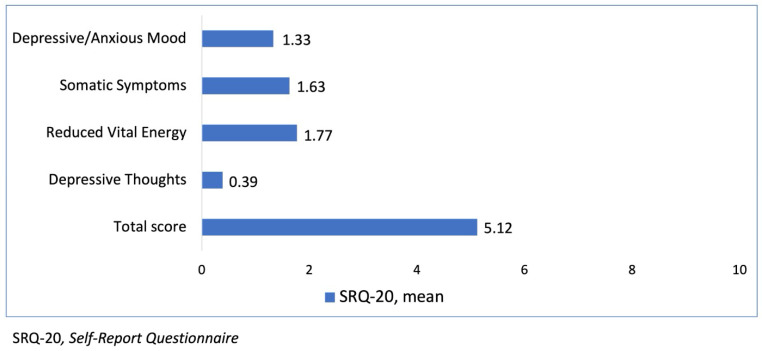
Mean scores of SRQ-20 (self-report questionnaire) domains (n = 203, São José do Rio Preto, Brazil, 2025). Mean values for SRQ-20 domains. SRQ-20, self-report questionnaire.

The mean scores for the different domains of the SRQ-20 were 1.33 for Depressive/Anxious Mood, 1.63 for Somatic Symptoms, 1.77 for Reduced Vital Energy, 0.39 for Depressive Thoughts, and the mean total score was 5.12—Figure 2.

Figure 2 graphically illustrates these mean scores.

### 3.3. Factor Analysis and Instrument Reliability

#### 3.3.1. Self-Report Questionnaire (SRQ-20)

The factorial structure of the SRQ-20 was evaluated through Bayesian confirmatory factor analysis, adopting a model of four correlated factors: Depressive–Anxious Mood (DAM), Somatic Symptoms (SS), Decreased Vital Energy (DEV), and Depressive Thoughts (DT). The Bayesian fit indices were considered adequate: the posterior predictive *p*-value (ppp) was 0.344; the Deviance Information Criterion (DIC) was 3327.168; the Watanabe-Akaike Information Criterion (WAIC) was 3343.535; and the Leave-One-Out Information Criterion (LOOIC) was 3343.781.

Table 1 presents the standardized factor loadings of the items for each of the four latent factors of the SRQ-20. These loadings demonstrate how strongly each item relates to its underlying factor, providing crucial evidence for the validity of the instrument’s factorial structure. All non-zero loadings are statistically significant, indicating a robust measurement model.

The correlations between the latent factors of the SRQ-20 were high and are presented in Table 2. This strong correlation among factors indicates that while distinct, these dimensions of minor mental disorders are highly intertwined.

The composite reliability for the Decreased Vital Energy (DEV) factor was 0.842, for Depressive–Anxious Mood (DAM) was 0.739, for Depressive Thoughts (DTs) was 0.826, and for Somatic Symptoms (SSs) was 0.762, indicating good to excellent internal consistency for all factors, thereby confirming their reliability in this sample.

#### 3.3.2. Wagnild and Young Resilience Scale

This study tested different models for the Wagnild and Young Resilience Scale. Table 3 compares the fit of Bayesian models for the 14- and 25-item versions, with and without collapsed response categories.

Table 4 presents the fit of these same models through the traditional confirmatory factor approach.

It was observed that categories 1, 2, 3, and 4 of the original 7-point scale had the lowest response rates and were collapsed into a single category, while categories 5, 6, and 7 became, respectively, 2, 3, and 4. Table 5 details the percentages of original responses in each category.

As indicated by the model fit indices in Table 3 and Table 4, the 14-item model with collapsed categories consistently presented the best balance of fit in both Bayesian and traditional confirmatory factor analysis approaches, validating its use for this study. The standardized factor loadings for this optimal model had a mean of 0.74 (SD = 0.08) and a median of 0.77. The composite reliability for the 14-item collapsed model was 0.860, indicating high internal consistency and supporting the robustness of this version of the scale in our sample.

#### 3.3.3. WHOQOL-BREF

The Bayesian factor analysis for WHOQOL-BREF evaluated Hierarchical, Partial Bifactor, and Complete Bifactor models. Table 6 presents the fit indices for these models.

The Complete Bifactor Model was the most adequate in terms of DIC (10,907.31), WAIC (11,043.11), and LOOIC (11,044.76). Table 7 details the factor loadings of this model.

The composite reliability for the Quality of Life (QoL) factor was 0.829, for the Physical factor was 0.561, for the Environmental factor was 0.266, and for the Social factor was 0.261. Critically, the Psychological factor presented extremely low composite reliability (0.00000983), indicating that the items associated with it do not significantly contribute to the measurement of the construct independently in this bifactor model. For this reason, the factor scores of the Psychological factor were not used in subsequent analyses.

### 3.4. Path Analysis Results

Path analysis was conducted to examine the predictive relationships between WHOQOL-BREF domains (General Quality of Life—QoL, Environmental—ENVIR, Physical—PHYS, Social—SOCIAL) and Resilience (RES) with dimensions of mental health (Decreased Vital Energy—DEV, Depressive–Anxious Mood—DAM, Depressive Thoughts—DTs, Somatic Symptoms—SSs). The analyses were based on 2000 posterior distributions of factor scores.

The fit indices of the path analysis models demonstrated excellent adequacy to the data: the mean chi-square value was 2.45 (SD = 1.36), with 12 fixed degrees of freedom and a mean *p*-value of 0.99 (SD = 0.03). The Comparative Fit Index (CFI) presented a mean of 1.00 (SD = 0), the Tucker–Lewis Index (TLI) a mean of 1.04 (SD = 0.01), and the Root Mean Square Error of Approximation (RMSEA) a mean of 0 (SD = 0), with a 95% confidence interval for the RMSEA reaching a maximum of 0.09.

Table 8 presents the descriptive statistics of the main results of the 2000 path analyses, including the standardized estimated values and *p*-values for the relationships between variables.

The results indicated that the overall Quality of Life (QoL) factor, derived from the WHOQOL-BREF after excluding the Psychological domain due to its inadequate reliability (as detailed in Section 3.3.3), was a robust and statistically significant predictor of all dimensions of mental health: Decreased Vital Energy (mean coef. = −0.579; *p* < 0.001), Depressive–Anxious Mood (mean coef. = −0.497; *p* < 0.001), Depressive Thoughts (mean coef. = −0.498; *p* < 0.001), and Somatic Symptoms (mean coef. = −0.523; *p* < 0.001). These strong negative relationships highlight the critical protective role of this comprehensive measure of overall QoL against mental health symptoms.

The Physical factor (PHYS) of WHOQOL-BREF also presented a significant role in predicting mental health variables, with regression coefficients ranging from −0.15 for DAM (*p* = 0.046) to −0.18 for SS (*p* = 0.024). In contrast, the Environmental (ENVIR) and Social (SOCIAL) variables of WHOQOL-BREF did not demonstrate a significant predictive role for mental health symptoms, with mean *p*-values above 0.3.

Resilience (RES) did not present a significant predictive role on mental health variables (high *p*-values, above 0.4) when the effect of quality of life was controlled. However, resilience correlated significantly with Quality of Life (QoL) (coef. = 0.515; *p* < 0.001). This suggests that, while resilience does not directly predict MMDs when QoL is accounted for, it maintains a strong positive association with QoL, indicating its potential indirect influence.

The mental health variables (DEV, SS, for DAM, and DT) presented relatively high explained variance (R^2^), with coefficients ranging from 0.586 to 0.695, indicating that between 30.5% and 41.4% of the variance in these variables was explained by the model’s predictors.

**Figure 3:**
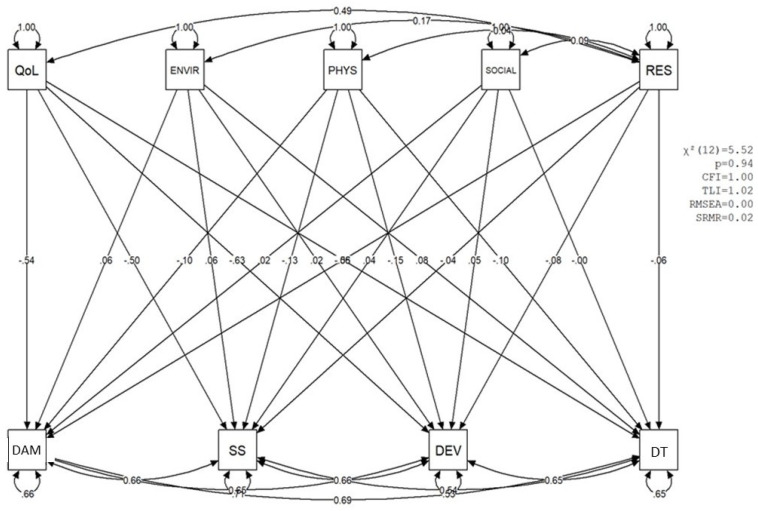
Visual representation of a path analysis model, including Quality of Life (QoL), Depressive–Anxious Mood (DAM), Somatic Symptoms (SSs), Decreased Vital Energy (DVE), and Depressive Thoughts (DTs) variables, with their respective standardized estimate values.

Figure 3 presents a visual example of one of the mental health prediction models in nurses, with their respective standardized estimate values.

### 3.5. Quality of Life and Minor Mental Disorders by Sociodemographic and Professional Data

This section presents a comparative analysis of Quality of Life (WHOQOL-Bref domains) and Minor Mental Disorders (SRQ-20) across various sociodemographic and professional characteristics of the sample. Statistically significant findings (*p* < 0.05) are highlighted within the tables for clarity. Detailed sociodemographic and professional characteristics of the participants are presented in Table 9 and Table 10.

## 4. Discussion

The mental health of nursing professionals is a critical global concern, and our study underscores the significant challenges faced by this essential workforce. This investigation aimed to delineate the prevalence of Minor Mental Disorders (MMDs) and resilience levels among nursing professionals, further exploring their intricate relationships with quality of life (QoL). Our findings highlight that a substantial proportion of nursing professionals experience MMDs, affirming the urgent need for targeted interventions. Crucially, we observed a strong relationship where a higher quality of life is strongly associated with fewer mental health symptoms, while resilience appears to bolster mental well-being indirectly through its positive influence on quality of life. These insights offer concrete directions for promoting the psychological well-being of nurses in demanding healthcare settings globally, emphasizing key protective factors and identifying areas for strategic support.

The prevalence of MMD in our sample, at 31.0%, is a compelling finding that aligns with a broad body of international literature identifying nursing as a risk group for mental health challenges [27,28,29]. For instance, studies from diverse Asian cohorts have consistently reported MMD prevalence rates ranging from 20% to over 40% among nurses, often exacerbated by factors such as demanding shift work, long working hours, and high patient loads [82]. Similarly, recent European studies underscore elevated rates of burnout, anxiety, and depression in nursing populations, particularly within high-pressure and under-resourced environments like emergency departments and intensive care units [83]. This percentage reflects the impact of demands inherent to the profession, such as excessive working hours, constant pressure, and exposure to critical situations, which characterize a challenging and stressful work environment [2,84]. The SRQ-20 domains with the highest mean scores were “Reduction of Vital Energy” and “Somatic Symptoms,” indicating significant physical and emotional exhaustion, aligned with the conceptualization of burnout as a phenomenon of exhaustion [13]. Occupational stress can lead to anxiety, depression, distress, poor performance, and hostility, outcomes that manifest in the observed MMDs.

A particularly robust and clinically relevant findings from our analysis is the consistent negative association observed between the overall General Quality of Life (QoL) (excluding the psychological domain due to its psychometric limitations, as discussed in Section 3.3.3) and all dimensions of MMD (Decreased Vital Energy, Depressive–Anxious Mood, Depressive Thoughts, and Somatic Symptoms), as presented in Table 8. This strong correlation indicates that a higher perceived quality of life is strongly associated with a lower presence and severity of MMD symptoms. It is crucial to emphasize that, given the cross-sectional design of this study, we cannot establish direct causal relationships; rather, these findings highlight a significant and consistent interplay where greater QoL co-occurs with better mental health outcomes. The observed relationships suggest that QoL acts as a critical factor in the overall mental well-being equation for nursing professionals, serving not merely as an indicator of well-being but as a potentially crucial protective element associated with the manifestation of mental health challenges. This emphasizes the importance of enhancing QoL as a strategic target for interventions. This result reinforces the tenets of the Biopsychosocial Model, which posits that an individual’s subjective perception of their life (QoL) interacts dynamically with psychological and social factors to profoundly influence mental health [18].

The role of resilience, while complex, emerged as a fundamental, albeit indirect, factor. While our initial hypothesis anticipated a direct predictive role of resilience on MMDs, particularly when controlling for QoL, the results did not support this direct link. Instead, we observed a highly significant and positive correlation between resilience and Quality of Life (QoL), as shown in Table 8. This intriguing pattern suggests that resilience may function as an indirect moderator or precursor to QoL, whereby QoL then exerts a more direct and robust influence on the reduction of MMDs. This finding resonates with emerging research from diverse international contexts; for instance, studies from European nurses indicate that, while resilience may not always directly mitigate distress, it consistently enhances coping efficacy and well-being, pathways that frequently lead to improved QoL [83]. Similarly, research among healthcare workers in Southeast Asia points to resilience as a crucial resource for maintaining a positive outlook and adaptive functioning, particularly by fostering a sense of control and self-efficacy that underpins better quality of life perceptions even amid high occupational stress [85,86]. In essence, individuals demonstrating greater resilience tend to report a higher perception of their quality of life, and this elevated QoL, in turn, appears to mediate the impact of resilience on mitigating MMD symptoms. This interpretation aligns well with the Transactional Model of Stress and Coping, where resilience, understood as a dynamic process that mobilizes adaptive coping strategies, contributes to a more constructive assessment of stressors. Consequently, this fosters better QoL, thereby minimizing the deleterious effects of stress on mental health [87]. Therefore, resilience is not merely the capacity to endure but to “positively rebuild,” manifested in the ability to maintain a satisfactory QoL even when confronted with adverse environments [32].

The analysis of the physical factor of WHOQOL-BREF as a significant correlate of MMDs suggests the inherent physical demands of nursing work, such as prolonged standing and significant physical exertion, are indeed linked to psychological well-being [15,88]. This finding underscores the necessity of integrated approaches that consider both the mental and physical dimensions of occupational health. Conversely, the Environmental and Social domains of WHOQOL-BREF did not demonstrate a significant association with mental health symptoms in our analysis (Table 8). While this non-significance could partly be attributed to the low composite reliability observed for these domains in our factor analysis of WHOQOL-BREF, suggesting a measurement artifact that likely obscured their true predictive or correlational role (as elaborated in Section 4.1, Limitations), it also prompts a broader consideration. In highly demanding and high-stress professional environments like nursing, the immediate and direct impacts of physical strain and psychological burden on mental health might be more pronounced and perceivable than the effects of perceived environmental comfort or social relationships. This is not to diminish the importance of supportive social and environmental factors, which are crucial for overall well-being and long-term sustainability. Instead, it suggests that in a context of acute and pervasive occupational stressors, their influence on MMDs might be more subtle or indirect, perhaps mediated through other constructs not fully captured by our current model (e.g., job satisfaction, organizational support culture, or individual coping styles related to social engagement). Future research employing qualitative methods or more granular, context-specific measures of these domains could provide deeper insights into their true influence, moving beyond purely psychometric considerations and exploring their nuanced roles in different work environments.

Our examination of sociodemographic and professional characteristics further revealed specific vulnerabilities within the nursing cohort, as detailed in Table 9 and Table 10. Notably, younger professionals (up to 39 years) and females exhibited a higher prevalence of MMDs and a lower perception of QoL. These data are consistent with existing literature suggesting increased susceptibility of these demographic groups to particular types of occupational stress, often compounded by the complex interplay of professional and personal responsibilities [26]. It is important to acknowledge that females comprised the overwhelming majority (81.3%) of our sample, which necessitates careful consideration when extrapolating these observed gender differences, as they may reflect the sample composition rather than intrinsic gender vulnerability alone.

To fully comprehend the observed interactions, it is crucial to consider the underlying biological and psychophysiological mechanisms. Chronic occupational stress can lead to dysregulation of the Hypothalamic–Pituitary–Adrenal (HPA) axis and the Autonomic Nervous System (ANS), manifesting in alterations in cortisol levels, reduced heart rate variability, and dysfunctions in neurotransmitter systems [39]. These neuroendocrine and neural alterations directly contribute to the symptoms of anxiety, depression, and fatigue that comprise MMDs. Conversely, resilience is associated with more effective regulation of these biological systems, facilitating faster recovery of homeostasis following stressful events and conferring protection against the deleterious effects of chronic stress [40]. This biological modulation by resilience is a fundamental aspect that underpins the protective role, albeit indirect, observed in our study. Our findings, suggesting the mediation of QoL, resonate with the need for integrated research that considers the various intrapsychic, interpersonal, familial, occupational, and social components, thereby supporting a holistic perspective on mental health.

### 4.1. Limitations

Despite the important contributions, this study has several limitations that should be considered in the interpretation of its results. First, the cross-sectional design inherently precludes the establishment of causal relationships between variables. Longitudinal studies are essential to explore the directionality of observed associations and the dynamic interplay of QoL, MMDs, and resilience over time.

Second, the use of self-report instruments (WHOQOL-BREF, SRQ-20, and Wagnild & Young Resilience Scale) may be subject to biases, such as social desirability bias, where participants may tend to provide socially acceptable responses.

Third, the low composite reliability of the Psychological, Environmental, and Social factors of WHOQOL-BREF in the complete bifactor model (with values of 0.00000983, 0.266, and 0.261, respectively) represents an important limitation. While the overall model fit was good, the inadequate measurement quality of these specific domains might have obscured their true predictive or correlational roles with mental health. The extremely low reliability of the Psychological factor, in particular, prevented its inclusion as an independent dimension in the path analysis, thus limiting specific conclusions about its unique contribution.

Finally, although we have discussed the underlying biological and psychophysiological mechanisms, the study did not include direct measurement of biomarkers (e.g., cortisol levels, heart rate variability). The inclusion of such measures could further strengthen the understanding of the complex interactions between psychosocial factors and the mental health of nursing professionals, providing a more solid empirical basis for discussions on the Biopsychosocial Model. Future research incorporating such direct biological measures would enhance the understanding of the complex interactions within the Biopsychosocial Model.

### 4.2. Implications

The results of this study have significant implications for clinical practice, occupational health management, and future research in the area of mental health of nursing professionals.

#### 4.2.1. Implications for Practice and Occupational Health Management

- Need for Comprehensive Interventions: The high prevalence of MMDs reinforces the urgency of mental health promotion programs and specific illness prevention for nursing professionals. These programs should go beyond simply detecting symptoms to address the roots of occupational stress.
- Focus on Quality of Life: Given the strong prediction of the overall QoL construct (as operationalized in this study) on MMDs, healthcare organizations should prioritize strategies that improve the quality of life of their professionals. This may include optimizing workloads, promoting a healthier and more collaborative work environment, offering adequate breaks, fostering work–life balance, and ensuring recognition and support.
- Resilience Programs: Although resilience did not directly predict MMDs in our model, its strong correlation with QoL suggests that developing resilience may be an effective indirect pathway to mitigate mental distress. Training programs in coping skills, emotional regulation, mindfulness, and promotion of social support networks can strengthen resilience and, consequently, improve QoL.
- Targeted Interventions: The greater vulnerability of younger professionals and females points to the need to develop interventions adapted to these demographics, considering their particularities and the specific challenges they face in the work environment and in their personal lives.
- Attention to Physical Aspects of Work: The physical domain of QoL being a predictor of MMDs underlines that working conditions that lead to physical wear should be reviewed. Ergonomic measures, more flexible work schedules, and the encouragement of physical health (e.g., physical activity, nutrition) can have a positive cascade impact on mental health.

#### 4.2.2. Implications for Future Research

- Longitudinal and Mediation Studies: Future research should employ longitudinal designs to establish causal relationships and investigate the mediating nature of QoL in the relationship between resilience and MMDs. More complex models can elucidate the dynamic interactions between these variables over time.
- Biological and Psychophysiological Mechanisms: Integrating the assessment of stress biomarkers (e.g., salivary cortisol, HRV) in future research can deepen the understanding of the underlying mechanisms that connect occupational stress, resilience, QoL, and MMDs, providing solid empirical evidence for the complex network of interactions.
- Development and Validation of Instruments: The low reliability of some domains of WHOQOL-BREF in our study points to the need to explore the adequacy of QoL assessment instruments and other constructs in specific populations of healthcare professionals, or to develop and validate new tools that better capture the nuance of these factors. Future research should particularly focus on validating and refining measures for the psychological, environmental, and social dimensions of quality of life within nursing contexts to ensure accurate and comprehensive assessment.
- Resilience and Engagement: Investigate how resilience not only mitigates suffering but also promotes engagement and professional fulfillment, connecting with the dimension of low personal accomplishment in burnout.
- Environmental and Social Factors in the Workplace: Despite the low reliability in the instrument, it is crucial to continue investigating the influence of social support in the work environment, organizational culture, and occupational health policies on the mental health of nurses, using methodologies that capture these complexities more effectively, especially considering the nuanced interplay suggested by our current findings.
- Focus on Quality of Life: Given the strong negative association/relationship of the overall QoL construct with MMDs, healthcare organizations should prioritize strategies that improve the quality of life of their professionals. This may include optimizing workloads, promoting a healthier and more collaborative work environment, offering adequate breaks, fostering work–life balance, and ensuring recognition and support.
- Attention to Physical Aspects of Work: The physical domain of QoL being associated with MMDs underlines that working conditions that lead to physical wear should be reviewed. Ergonomic measures, more flexible work schedules, and the encouragement of physical health (e.g., physical activity, nutrition) can have a positive cascade impact on mental health.

## 5. Conclusions

This study confirmed the high prevalence of Minor Mental Disorders among nursing professionals, reinforcing the urgency of attention to their mental health. We demonstrated that the overall Quality of Life (as assessed by the robust dimensions of the WHOQOL-BREF) is robustly and significantly associated with a lower prevalence and severity of these disorders, while resilience strongly correlates with QoL, suggesting an indirect protective role via enhancement of well-being perception.

Our findings underscore the importance of an integrated approach that considers the overall QoL as a primary target for occupational health interventions, while promoting resilience as a valuable individual resource. Understanding the interconnection between occupational stressors, psychosocial, and biological mechanisms is fundamental to developing more effective and targeted support strategies. By contributing to a more holistic understanding of mental health in nursing, this work aims to support the formulation of health promotion measures that ensure the well-being of professionals who are the foundation of our healthcare systems.

## Figures and Tables

**Table 1:** Standardized factor loadings of the Bayesian confirmatory factor analysis of SRQ-20. (n = 203, São José do Rio Preto, Brazil, 2025).

Items	Factors			
	DAM	SS	DEV	DT
SRQ4	0.527	0.000	0.000	0.000
SRQ6	0.842	0.000	0.000	0.000
SRQ9	0.890	0.000	0.000	0.000
SRQ10	0.862	0.000	0.000	0.000
SRQ1	0.000	0.525	0.000	0.000
SRQ2	0.000	0.723	0.000	0.000
SRQ3	0.000	0.806	0.000	0.000
SRQ5	0.000	0.457	0.000	0.000
SRQ7	0.000	0.874	0.000	0.000
SRQ19	0.000	0.913	0.000	0.000
SRQ8	0.000	0.000	0.672	0.000
SRQ11	0.000	0.000	0.811	0.000
SRQ12	0.000	0.000	0.666	0.000
SRQ13	0.000	0.000	0.679	0.000
SRQ18	0.000	0.000	0.951	0.000
SRQ20	0.000	0.000	0.933	0.000
SRQ14	0.000	0.000	0.000	0.531
SRQ15	0.000	0.000	0.000	0.950
SRQ16	0.000	0.000	0.000	0.907
SRQ17	0.000	0.000	0.000	0.867

DAM: Depressive–Anxious Mood; SS: Somatic Symptoms; DEV: Decreased Vital Energy; DT: Depressive Thoughts. All non-zero factor loadings are statistically significant.

**Table 2:** Correlation of factors from the Bayesian confirmatory factor analysis of SRQ-20. (n = 203, São José do Rio Preto, Brazil, 2025).

Factors	DAM	SS	DEV	DT
DAM	1.000	0.760	0.758	0.783
SS	0.760	1.000	0.782	0.674
DEV	0.758	0.782	1.000	0.764
DT	0.783	0.674	0.764	1.000

DAM: Depressive-Anxious Mood; SS: Somatic Symptoms; DEV: Decreased Vital Energy; DT: Depressive Thoughts. All correlations are statistically significant (*p* < 0.001).

**Table 3:** Fit of Bayesian factor models of the resilience scale.

Model	Ppp	dic	WAIC	LOOIC
RS—14 items	0.000	7696.209	7710.945	7711.982
RS—14 items collapsed	0.003	6228.889	6237.308	6237.537
RS—25 items	0.000	14,595.58	14,629.50	14,629.45
RS—25 items collapsed	0.000	13,157.45	13,181.79	13,182.28

**Table 4:** Fit of confirmatory factor models of the resilience scale.

Model	Chisq	df	*p* Value	cfi	tli	rmsea [95% CI]
RS—14 items	236.758	77	<0.001	0.982	0.979	0.101 [0.087; 0.116]
RS—14 items collapsed	142.546	77	<0.001	0.991	0.990	0.065 [0.048; 0.081]
RS—25 items	679.266	265	<0.001	0.977	0.974	0.088 [0.080; 0.096]
RS—25 items collapsed	575.463	265	<0.001	0.983	0.981	0.076 [0.068; 0.085]

**Table 5:** Percentages of responses by category in the Wagnild and Young resilience scale (203 observations).

Items	Response Categories						
	1	2	3	4	5	6	7
R1	2.46	1.97	7.88	19.70	17.24	18.72	32.02
R2	0.49	0.49	5.42	8.87	24.63	25.12	34.98
R3	0.99	0.99	4.43	12.81	15.76	27.59	37.44
R4	1.48	0.49	3.94	7.88	18.72	22.17	45.32
R5	2.46	1.97	4.93	14.78	17.24	22.17	36.45
R6	0.00	0.49	1.48	4.93	12.32	21.67	59.11
R7	12.32	7.39	12.32	23.15	19.21	9.36	16.26
R8	2.46	3.94	4.43	11.33	12.32	14.78	50.74
R9	4.93	2.46	3.94	12.81	21.18	24.63	30.05
R10	0.49	2.96	4.43	13.30	12.32	24.14	42.36
R11	24.63	17.24	14.29	13.30	11.33	6.90	12.32
R12	6.40	7.88	12.81	22.17	15.27	16.26	19.21
R13	0.49	0.99	5.91	13.79	15.27	21.18	42.36
R14	0.99	3.45	9.36	14.29	14.78	24.63	32.51
R15	0.99	1.48	4.93	17.24	16.75	28.57	30.05
R16	1.97	0.99	5.91	13.30	18.72	24.14	34.98
R17	1.97	3.94	4.43	13.79	14.78	20.20	40.89
R18	0.99	0.00	1.48	4.93	10.84	24.14	57.64
R19	0.99	0.49	1.97	11.82	19.21	22.66	42.86
R20	3.94	4.93	5.91	15.76	17.73	23.15	28.57
R21	1.48	0.49	2.96	4.93	8.37	20.69	61.08
R22	7.88	5.42	8.87	17.24	18.72	21.67	20.20
R23	0.99	0.00	3.94	14.29	17.24	29.56	33.99
R24	3.94	4.43	7.88	18.23	16.26	22.17	27.09
R25	0.99	2.96	6.40	12.32	11.33	23.15	42.86

Columns from 1 to 7 represent the original response categories of the scale.

**Table 6:** Fit of Bayesian factor models of WHOQOL-BREF.

Model	Ppp	dic	WAIC	LOOIC
Hierarchical Model	0.000	11,076.69	11,097.83	11,098.10
Partial Bifactor Model	0.000	10,962.22	11,044.00	11,045.48
Complete Bifactor Model	0.000	10,907.31	11,043.11	11,044.76

**Table 7:** Standardized factor loadings of the Bayesian confirmatory factor analysis of WHOQOL-BREF (complete bifactor model).

Items	Factors				
	Physical	Psychological	Social	Environmental	QoL
WB17	0.170	0.000	0.000	0.000	0.740
WB3	0.885	0.000	0.000	0.000	0.312
WB4	0.757	0.000	0.000	0.000	0.337
WB10	0.135	0.000	0.000	0.000	0.767
WB15	0.355	0.000	0.000	0.000	0.333
WB16	0.166	0.000	0.000	0.000	0.643
WB18	0.313	0.000	0.000	0.000	0.697
WB19	0.000	0.033	0.000	0.000	0.842
WB5	0.000	−0.026	0.000	0.000	0.587
WB6	0.000	−0.027	0.000	0.000	0.644
WB7	0.000	−0.076	0.000	0.000	0.610
WB11	0.000	0.057	0.000	0.000	0.606
WB26	0.000	0.004	0.000	0.000	0.368
WB20	0.000	0.000	0.326	0.000	0.647
WB21	0.000	0.000	0.120	0.000	0.612
WB22	0.000	0.000	0.749	0.000	0.558
WB8	0.000	0.000	0.000	0.055	0.728
WB9	0.000	0.000	0.000	0.133	0.490
WB12	0.000	0.000	0.000	0.087	0.506
WB13	0.000	0.000	0.000	0.184	0.457
WB14	0.000	0.000	0.000	0.032	0.531
WB23	0.000	0.000	0.000	0.457	0.495
WB24	0.000	0.000	0.000	0.510	0.481
WB25	0.000	0.000	0.000	0.799	0.440

QoL: General Quality of Life. All non-zero factor loadings are statistically significant.

**Table 8:** Descriptive statistics of the main results of the 2000 path analyses.

Relationship of Variables			Distribution of Standardized Estimated Values						*p*-Value	
lhs	op	Rhs	Mn	P25	P50	Mean	P75	Mx	Mean	SD
WHOQOL-BREF Variables										
DEV	~	QoL	−0.691	−0.604	−0.580	−0.579	−0.557	−0.463	<0.001	<0.001
DAM	~	QoL	−0.611	−0.523	−0.499	−0.497	−0.471	−0.320	<0.001	<0.001
DT	~	QoL	−0.635	−0.526	−0.497	−0.498	−0.471	−0.345	<0.001	<0.001
SS	~	QoL	−0.645	−0.549	−0.524	−0.523	−0.498	−0.395	<0.001	<0.001
DEV	~	ENVIR	−0.114	0.012	0.045	0.046	0.080	0.198	0.408	0.302
DAM	~	ENVIR	−0.110	0.021	0.051	0.053	0.085	0.217	0.399	0.291
DT	~	ENVIR	−0.137	0.007	0.040	0.041	0.075	0.239	0.449	0.296
SS	~	ENVIR	−0.111	0.008	0.040	0.041	0.074	0.215	0.449	0.296
DEV	~	PHYS	−0.355	−0.199	−0.165	−0.163	−0.128	−0.007	0.034	0.090
DAM	~	PHYS	−0.356	−0.191	−0.156	−0.155	−0.120	0.065	0.046	0.111
DT	~	PHYS	−0.317	−0.167	−0.133	−0.131	−0.097	0.062	0.089	0.165
SS	~	PHYS	−0.341	−0.211	−0.179	−0.177	−0.143	0.016	0.024	0.075
DEV	~	SOCIAL	−0.158	0.018	0.053	0.052	0.088	0.216	0.380	0.303
DAM	~	SOCIAL	−0.158	−0.009	0.027	0.028	0.062	0.206	0.488	0.291
DT	~	SOCIAL	−0.167	−0.006	0.032	0.031	0.068	0.233	0.467	0.289
SS	~	SOCIAL	−0.216	−0.023	0.014	0.013	0.048	0.196	0.514	0.284
Wagnild and Young Resilience Scale Variables, 14-item version										
DEV	~	RES	−0.205	−0.088	−0.058	−0.058	−0.026	0.079	0.437	0.280
DAM	~	RES	−0.228	−0.075	−0.042	−0.043	−0.011	0.112	0.525	0.273
DT	~	RES	−0.224	−0.084	−0.048	−0.047	−0.012	0.140	0.495	0.280
SS	~	RES	−0.165	−0.047	−0.014	−0.012	0.021	0.164	0.614	0.246
ENVIR	~~	RES	−0.070	0.044	0.075	0.075	0.105	0.205	0.282	0.271
PHYS	~~	RES	−0.173	−0.026	0.005	0.006	0.038	0.184	0.555	0.277
QoL	~~	RES	0.436	0.497	0.515	0.515	0.532	0.594	<0.001	<0.001
SOCIAL	~~	RES	−0.134	0.006	0.041	0.039	0.075	0.194	0.457	0.292
Variance of model variables										
DEV	~~	DEV	0.505	0.569	0.586	0.586	0.604	0.679	<0.001	<0.001
SS	~~	SS	0.590	0.659	0.676	0.676	0.693	0.760	<0.001	<0.001
DAM	~~	HuDA	0.597	0.676	0.692	0.692	0.708	0.787	<0.001	<0.001
PD	~~	PD	0.601	0.677	0.695	0.695	0.712	0.793	<0.001	<0.001

lhs: left-hand side; op: operator; rhs: right-hand side; ~: Regression (or prediction); ~~: Correlation or covariance; Mn: Minimum; P25: 25th Percentile; P50: 50th Percentile; P75: 75th Percentile; Mx: Maximum; SD: Standard Deviation; QoL: Quality of Life; ENVIR: Environmental; DEV: Decreased Vital Energy; DAM: Depressive–Anxious Mood; DTs: Depressive Thoughts; SSs: Somatic Symptoms; RES: Resilience.

**Table 9:** Comparative analysis of Quality of Life (WHOQOL-Bref Domains) according to sociodemographic (**A**) and professional (**B**) data. (n = 203, São José do Rio Preto, Brazil, 2025).

(A) Sociodemographic Data								
Variable	WHOQOL-Bref Domain							
	Physical	*p* Value	Psychological	*p* Value	Social relations	*p* Value	Environment	*p* Value
Age, n (%)								
<=39 years	71.4	0.207	66.7	0.026	66.7	0.004	65.6	0.699
>39 years	71.4		70.8		75.0		62.5	
Sex, n (%)								
Female	67.9	0.049	66.7	0.007	66.7	0.005	62.5	0.602
Male	75.0		72.9		76.0		64.1	
Marital status, n (%)								
With partner	71.4	0.391	66.7	0.686	66.7	0.353	62.5	0.997
Without partner	71.4		66.7		66.7		65.6	
Number of children, n (%)								
None	71.4	0.238	66.7	0.909	66.7	0.848	65.6	0.492
One or more children	67.9		66.7		66.7		62.5	
Family income in reais, n (%)								
<=1000	57.1	0.186	50.0	**0.026**	50.0	0.314	59.4	0.755
From 1001 to 3000	71.4		75.0		66.7		64.1	
>=3000	69.6		66.7		66.7		62.5	
**(B) Professional Data**								
Variable	WHOQOL-Bref Domain							
	Physical	*p* Value	Psychological	*p* Value	Social relations	*p* Value	Environment	*p* Value
Working hours, n (%)								
30 h weekly	71.4	0.368	70.8	0.296	75.0	0.187	68.8	**0.021**
From 31 to 44 h weekly	71.4		66.7		66.7		64.1	
More than 44 h weekly	67.9		66.7		50.0		53.1	
Number of employment relationships, n (%)								
One	71.4	**0.025**	66.7	0.234	66.7	0.205	62.5	0.067
Two	67.9		66.7		66.7		62.5	
Three	91.1		77.1		79.2		76.6	
Work shift, n (%)								
Daytime	71.4	0.125	70.8	0.141	66.7	0.618	62.5	0.786
Nighttime	67.9		66.7		66.7		62.5	
More than one shift	71.4		66.7		66.7		65.6	
Academic qualification, n (%)								
Specialization	71.4	0.411	66.7	0.219	66.7	0.130	65.6	0.473
Master’s/Doctorate	57.1		58.3		58.3		62.5	
Work sector, n (%)								
Emergency	71.4	0.139	70.8	0.118	66.7	0.064	62.5	0.982
Inpatient units	67.9		66.7		66.7		65.6	

Legend: Values in percentage (n%) and respective *p*-values. Statistically significant *p*-values (*p* < 0.05) are highlighted in bold.

**Table 10:** Comparative analysis of minor mental disorders (SRQ-20) according to sociodemographic (**A**) and professional (**B**) data.

(A) Sociodemographic Data										
Variable	Minor Mental Disorders, According to SRQ									
	SRQ total	*p* Value	Depressive/anxious mood	*p* Value	Somatic symptoms	*p* Value	Reduced vital energy	*p* Value	Depressive thoughts	*p* Value
Age, n (%)										
<=39 years	6.0	**0.012**	1.0	**0.009**	1.0	0.116	2.0	**0.017**	0.0	0.224
>39 years	3.0		1.0		1.0		1.0		0.0	
Sex, n (%)										
Female	5.0	**0.023**	1.0	**0.009**	1.0	**0.041**	1.0	0.104	0.0	0.160
Male	2.0		0.5		0.5		0.5		0.0	
Marital status, n (%)										
With partner	4.0	0.846	1.0	0.619	1.0	0.549	1.0	0.934	0.0	0.612
Without partner	5.0		1.0		1.0		1.0		0.0	
Number of children, n (%)										
None	5.0	0.836	1.0	0.902	1.0	0.796	1.0	0.908	0.0	0.882
One or more children	4.0		1.0		1.0		1.0		0.0	
Family income in reais, n (%)										
<=1000	10.0	0.146	1.0	0.071	3.0	0.229	3.0	0.285	0.0	0.576
1001 to 3000	3.0		1.0		1.0		1.0		0.0	
>=3000	5.0		1.0		1.0		1.0		0.0	
**(B) Professional Data**										
**Variable**	**Minor Mental Disorders, According to SRQ**									
	SRQ total	*p* Value	Depressive/anxious mood	*p* Value	Somatic symptoms	*p* Value	Reduced vital energy	*p* Value	Depressive thoughts	*p* Value
Working hours, n (%)										
30 h weekly	3.5	0.514	1.0	0.768	1.0	0.225	1.0	0.263	0.0	0.375
From 31 to 44 h weekly	5.0		1.0		1.0		1.0		0.0	
More than 44 h weekly	5.0		1.0		1.0		2.0		0.0	
Number of employment relationships, n (%)										
One	5.0	0.064	1.0	0.128	1.0	0.134	1.0	0.074	0.0	0.504
Two	4.5		1.0		1.0		2.0		0.0	
Three	0.0		0.0		0.0		0.0		0.0	
Work shift, n (%)										
Daytime	4.0	0.587	1.0	0.256	1.0	0.334	1.0	0.689	0.0	0.065
Nighttime	5.0		1.0		2.0		1.0		0.0	
More than one shift	4.0		1.0		1.0		2.0		0.0	
Academic qualification, n (%)										
Specialization	5.0	0.628	1.0	0.751	2.0	0.210	1.5	0.084	0.0	0.864
Master’s/Doctorate	6.0		2.0		1.0		3.0		0.0	
Work sector, n (%)										
Emergency	4.0	0.137	1.0	0.232	1.0	0.211	1.0	0.233	0.0	0.768
Inpatient units	5.0		1.0		2.0		2.0		0.0	

Values (n%) and respective *p*-values. SRQ total: total score of the Self-Report Questionnaire. Depressive/anxious mood, Somatic symptoms, Reduced vital energy, Depressive thoughts: SRQ domains. (n = 203, São José do Rio Preto, Brazil, 2025). Statistically significant *p*-values (*p* < 0.05) are highlighted in bold.

## Data Availability

Data are contained within the article or Supplementary Materials. Further inquiries can be directed to the corresponding author.

## Supplementary Materials

The following supporting information can be downloaded at: https://www.mdpi.com/article/10.3390/ijerph22091375/s1, Supplementary File S1: Strengthening the Reporting of Observational Studies in Epidemiology (STROBE) guidelines.

## Abbreviations

The following abbreviations are used in this manuscript:

ANS	Autonomic Nervous System
CAAE	Certificate of Presentation for Ethical Appraisal
CFI	Comparative Fit Index
DAM	Depressive–Anxious Mood
DIC	Deviance Information Criterion
DT	Depressive thoughts
FAMERP	Faculty of Medicine of São José do Rio Preto
GAS	General Adaptation Syndrome
HPA	Hypothalamic-Pituitary-Adrenal
JD-R	Job Demands-Resources
LOOIC	Leave-One-Out Information Criterion
MMD	Minor Mental Disorders
QoL	Quality of Life
RMSEA	Root Mean Square Error of Approximation
SRQ-20	Self-Report Questionnaire
SS	Somatic Symptoms
TLI	Tucker-Lewis Index
UPAs	Emergency Care Units
WAIC	Watanabe-Akaike Information Criterion
WHO	World Health Organization

## References

[1] Fricke J., Siddique S.M., Douma C., Ladak A., Burchill C.N., Greysen R., Mull N.K. (2023). Workplace Violence in Healthcare Settings: A Scoping Review of Guidelines and Systematic Reviews. Trauma Violence Abus..

[2] Crawford C.L., Chu F., Judson L.H., Cuenca E., Jadalla A.A., Tze-Polo L., Kawar L.N., Runnels C., Garvida R. (2019). An Integrative Review of Nurse-to-Nurse Incivility, Hostility, and Workplace Violence. Nurs. Adm. Q..

[3] Anusiewicz C.V., Ivankova N.V., Swiger P.A., Gillespie G.L., Li P., Patrician P.A. (2020). How does workplace bullying influence nurses’ abilities to provide patient care? A nurse perspective. J. Clin. Nurs..

[4] Pogue C.A., Li P., Swiger P., Gillespie G., Ivankova N., Patrician P.A. (2022). Associations among the nursing work environment, nurse-reported workplace bullying, and patient outcomes. Nurs. Forum.

[5] Souza J.S., Reis E.A., Godman B., Campbell S.M., Meyer J.C., Sena L.W.P., Godói I.P.D. (2024). Users’ Perceptions of Access to and Quality of Unified Health System Services in Brazil: A Cross-Sectional Study and Implications to Healthcare Management Challenges. Int. J. Environ. Res. Public Health.

[6] Saxena S., Rathore B. (2025). Adversity Quotient as Determining Factor of Mental Health and Professional Quality of Life Among Healthcare Professionals: A Systematic Review. Ann. Neurosci..

[7] Selye H. (1936). A Syndrome produced by Diverse Nocuous Agents. Nature.

[8] Vieira G.C., Granadeiro Dda S., Raimundo D.D., Da Silva J.F., Hanzelmann Rda S., Passos J.P. (2021). Satisfação profissional e qualidade de vida de enfermeiros de um hospital brasileiro. Av. Enfermería.

[9] Mallagoli I.S.S., da Silva E.P., Oliveira M.A.D.N., Barbosa I.E.B., Sampaio A.N., Matias A.B., Barbosa D.A., Belasco A.G.S. (2024). Quality of life associated with nursing professionals’ individual resources and work. Rev. Bras. Enferm..

[10] De Santos C.C.A., Gomes N.R., Santos K.O.B., de Medeiros A.M. (2024). Avaliação dos aspectos psicossociais do trabalho no Brasil no contexto da saúde do trabalhador: Uma revisão de escopo. Rev. Bras. Saúde Ocup..

[11] Demerouti E., Bakker A.B., Nachreiner F., Schaufeli W.B. (2001). The job demands-resources model of burnout. J. Appl. Psychol..

[12] de Assis B.B., Azevedo C., de Moura C.C., Mendes P.G., Rocha L.L., Roncalli A.A., Vieira N.F.M., Chianca T.C.M. (2022). Factors associated with stress, anxiety and depression in nursing professionals in the hospital context. Rev. Bras. Enferm..

[13] Maslach C., Jackson S.E. (1981). The measurement of experienced burnout. J. Organ. Behav..

[14] Kiymaz D., Koç Z. (2023). Workplace violence, occupational commitment and intention among emergency room nurses: A mixed-methods study. J. Clin. Nurs..

[15] Makiyama M., Rizzotto M.L.F., Nasi C., Zack B.T., Machineski G.G. (2023). Práticas de saúde mental na atenção básica sob a ótica dos profissionais gestores. Rev. Baiana Enferm..

[16] Vega E.A.U., Macedo A.B.T., Antoniolli L., Pinheiro J.M.G., Esteban A.N.P., de Souza S.B.C. (2023). Levels of Anxiety and Stress Experienced by Nurses in Inpatient Units. Aquichan.

[17] Soder R.M., Escobar Dos Santos L., Cristine Oliveira I., Anacleto da Silva L.A., Cechinel Peiter C., Guedes dos Santos J.L. (2020). Práticas de enfermeiros na gestão do cuidado na atenção básica. Rev. Cubana Enferm..

[18] Engel G.L. (1977). The Need for a New Medical Model: A Challenge for Biomedicine. Science.

[19] The Whoqol Group (1998). Development of the World Health Organization WHOQOL-BREF Quality of Life Assessment. Psychol Med..

[20] Fleck M.P., Louzada S., Xavier M., Chachamovich E., Vieira G., Santos L., Pinzon V. (2000). Aplicação da versão em português do instrumento abreviado de avaliação da qualidade de vida “WHOQOL-bref”. Rev. Saude Publica.

[21] Dos Santos E.R., Bertolin D.C., dos Santos L.L., dos Santos Júnior R., da Silva P., de Almeida Sasso L.S., André J.C., da Fucuta P.S. (2020). Quality of life and resilience in nursing staff working in hospitalization units and emergency departments. Int. J. Dev. Res..

[22] Da Silveira R.C.P., da Ribeiro I., Mininel V.A. (2021). Qualidade de vida, perfil sociodemografico e laboral da equipe de enfermagem de um hospital universitário. Enfermería Actual Costa Rica.

[23] De Lima L.A.O., Silva L.L., Domingues Júnior P.L. (2024). Qualidade de Vida no Trabalho segundo as percepções dos funcionários públicos de uma Unidade Básica de Saúde (UBS). Rev. Carreiras Pessoas.

[24] Hernandes L.F., Gonçalves J.A., Da Silva W.C. (2023). Entre o adoecimento e trabalho: Ações de qualidade de vida no âmbito de saúde mental. Arq. Ciências Saúde UNIPAR.

[25] Nunes T.D., Torres A.C.S. (2023). Work process and mental health: Perceptions of workers at a Psychosocial Care Center. Res. Soc. Dev..

[26] Morsy M.M.E., Ebraheem S.M.A. (2020). Work-Related Stressors, Coping Strategies: Its Relation to Job Performance and Perceived Organizational Support among Critical Care Nurses. Evid.Based Nurs. Res..

[27] De Santana L., Ramos T.H., Haeffner R., Brey C., Pedrolo E., Ziesemer Nde B. (2024). Prevalência e fatores associados aos transtornos mentais e comportamentais entre trabalhadoras/es de enfermagem. Rev. Gaúcha Enferm..

[28] Castillo-González A., Velando-Soriano A., de La Fuente-Solana E.I., Martos-Cabrera B.M., Membrive-Jiménez M.J., Lucía R., La Fuente G.A.C. (2024). Relation and effect of resilience on burnout in nurses: A literature review and meta-analysis. Int. Nurs. Rev..

[29] Pereira M.C., Eberhardt L.D., de Carvalho M. (2024). Working conditions and mental illness among nursing workers. Rev. Bras. Med. Trab..

[30] Gonçalves D.M., Stein A.T., Kapczinski F. (2008). Avaliação de desempenho do Self-Reporting Questionnaire como instrumento de rastreamento psiquiátrico: Um estudo comparativo com o Structured Clinical Interview for DSM-IV-TR. Cad. Saude Publica.

[31] De Jesus Mari J., Williams P. (1986). A Validity Study of a Psychiatric Screening Questionnaire (SRQ-20) in Primary Care in the city of Sao Paulo. Br. J. Psychiatry.

[32] Bottini F.F., de Paiva K.C.M., de Gomes R.C. (2021). Resiliência individual, prazer e sofrimento no trabalho e vínculos organizacionais: Reflexões e perspectivas de pesquisas para o setor público. Cad EBAPEBR.

[33] Cunha I.C.K.O. (2020). Resiliência: Uma competência da Enfermagem. Enferm. Foco.

[34] Pousa P.C.P., de Lucca S.R. (2021). Psychosocial factors in nursing work and occupational risks: A systematic review. Rev. Bras. Enferm..

[35] De Medeiros S.E.G., de Aquino J.M., de Arruda G.A., do Robazzi M.L.C.C., da Gomes B.M.R., Andrade M.S., Monteiro E.M.L.M. (2023). Estresse e sofrimento em enfermeiros hospitalares: Relação com variáveis pessoais, laborais e hábitos de vida. Texto. Context Enferm..

[36] Sullivan V., Hughes V., Wilson D.R. (2022). Nursing Burnout and Its Impact on Health. Nurs. Clin. North Am..

[37] Moraes Filho I.M.D., Nascimento F.A.D., Bastos G.P., Barros Júnior F.E.D.S., Silva R.M.D., Santos A.L.M., Valóta I.A.D.C. (2020). Fatores sociodemográficos e acadêmicos relacionados à resiliência dos graduandos da área da saúde. REVISA.

[38] Seligman M.E.P., Csikszentmihalyi M. (2000). Positive psychology: An introduction. Am. Psychol..

[39] McEwen B.S. (2007). Physiology and Neurobiology of Stress and Adaptation: Central Role of the Brain. Physiol Rev..

[40] Miller A.H., Maletic V., Raison C.L. (2009). Inflammation and Its Discontents: The Role of Cytokines in the Pathophysiology of Major Depression. Biol. Psychiatry.

[41] Schultz C.C., de Colet C.F., Benetti E.R.R., Tavares J.P., Stumm E.M.F., Treviso P. (2022). A resiliência e a redução do estresse ocupacional na Enfermagem. Rev. Lat. Am. Enfermagem..

[42] Liao L., Wu Q., Su Y., Li R., Wang L. (2025). Coping Styles Mediated the Association Between Perceived Organizational Support and Resilience in Emergency Nurses Exposed to Workplace Violence: A Cross-Sectional Study. Nurs. Health Sci..

[43] Yang J., Chen Y., Tian Y., Li X., Yu Q., Huang C., Chen Z., Ning M., Li S., He J. (2024). Risk factors and consequences of mental health problems in nurses: A scoping review of cohort studies. Int. J. Ment. Health Nurs..

[44] Do Vale M.A., Biasi E.Y., de Lucca S.R. (2025). Psychosocial factors at work and common mental disorders in health care professionals and workers. Rev. Bras. Med. Trab..

[45] Huey C.W.T., Palaganas J.C. (2020). What are the factors affecting resilience in health professionals? A synthesis of systematic reviews. Med. Teach..

[46] Leonti R.M., Turliuc M.N. (2025). Better and Healthier Together? The Mediation Effect of Positive Psychological Capital on the Relationship Between Perceived Social Support and Health-Related Quality of Life Among Older Adults. Int. J. Aging Hum. Dev..

[47] Hospital de Base de São José do Rio Preto. https://www.hospitaldebase.com.br/.

[48] Hidalgo-Rasmussen C., Morales G., Ortiz M., Rojas M., Balboa-Castillo T., Lanuza F., Muñoz S. (2021). Propiedades psicométricas de la versión chilena del Whoqol-Bref para la calidad de vida. Behav. Psychol. Conduct..

[49] Beusenberg M., Orley J.H., World Health Organization (1994). A User’s Guide to the Self Reporting Questionnaire (SRQ/Compiled by, M. Beusenberg and J. Orley. https://iris.who.int/handle/10665/61113.

[50] Harding T.W., Climent C.E., Diop M.B., Giel R., Ibrahim H.H., Murthy R.S., Suleiman M.A., Wig N.N. (1983). The WHO collaborative study on strategies for extending mental health care, II: The development of new research methods. Am. J. Psychiatry.

[51] Iacoponi E., Jair de Jesus M. (1989). Reliability and Factor Structure of the Portuguese Version of Self-Reporting Questionnaire. Int. J. Soc. Psychiatry.

[52] Santos K.O.B., de Araújo T.M., de Oliveira N.F. (2009). Estrutura fatorial e consistência interna do Self-Reporting Questionnaire (SRQ-20) em população urbana. Cad. Saude Publica.

[53] Oliveira Bernardes Santos K., Araújo T.M., de Sousa Pinho P., de Conceição Silva A.C. (1970). Avaliação de um instrumento de mensuração de morbidade psíquica: Estudo de validação do Self-Reporting Questionnaire (SRQ-20). Rev Baiana Saúde Pública.

[54] Scazufca M., Menezes P.R., Vallada H., Araya R. (2009). Validity of the self reporting questionnaire-20 in epidemiological studies with older adults. Soc. Psychiatry Psychiatr. Epidemiol..

[55] Wagnild G.M., Young H.M. (1993). Development and Psychometric Evaluation of the Resilience Scale. J. Nurs. Meas..

[56] Pesce R.P., Assis S.G., Avanci J.Q., Santos N.C., Malaquias J.V., Carvalhaes R. (2005). Adaptação transcultural, confiabilidade e validade da escala de resiliência. Cad. Saude Publica.

[57] Perim P.C., Dias C.S., Corte-Real N.J., Andrade A.L., Fonseca A.M. (2015). Análise fatorial confirmatória da versão Brasileira da Escala de Resiliência (ER-Brasil). Gerais Rev. Interinstitucional Psicol..

[58] DiStefano C., Shi D., Morgan G.B. (2021). Collapsing Categories is Often More Advantageous than Modeling Sparse Data: Investigations in the CFA Framework. Struct. Equ. Model A Multidiscip. J..

[59] Nering M.L. (2010). Handbook of Polytomous Item Response Theory Models-Google Livros. https://books.google.com.br/books?hl=ptBR&lr=&id=4Yd7jSDxsc8C&oi=fnd&pg=PP1&dq=Handbook+of+polytomous+item+response+theory+models&ots=X_yj2HvREA&sig=VJMJh4-u8zIjNtYRR5959y_Pxe8#v=onepage&q=Handbookofpolytomousitemresponsetheorymodels&f=false.

[60] Reise S.P., Revicki D.A. (2014). Handbook of Item Response Theory Modeling.

[61] R Development Core Team (2020). RA Language and Environment for Statistical Computing.

[62] Farias H.B., Gomes C.M.A., Jelihovschi E.G. (2024). A new methodology to evaluate factor scores: Internal and external correlational accuracy. Int. J. Educ. Res..

[63] Kaplan D., Depaoli S., Hoyle H. (2012). Bayesian structural equation modeling. Handbook of Structural Equation Modeling.

[64] Muthén B., Asparouhov T. (2012). Bayesian structural equation modeling: A more flexible representation of substantive theory. Psychol. Methods.

[65] Merkle E.C., Rosseel Y. (2018). blavaan: Bayesian Structural Equation Models via Parameter Expansion. J. Stat. Softw..

[66] Gelman A., Carlin J.B., Stern H.S., Rubin D.B. (2003). Bayesian Data Analysis.

[67] Muthén B., Muthén L.K., Asparouhov T. (2010). Bayesian Analysis Using Mplus. https://scispace.com/pdf/bayesian-analysis-using-mplus-3yyuz79m2g.pdf.

[68] Vehtari A., Gelman A., Gabry J. (2017). Practical Bayesian model evaluation using leave-one-out cross-validation and WAIC. Stat. Comput..

[69] Yung Y.F., Thissen D., McLeod L.D. (1999). On the Relationship between the Higher-Order Factor Model and the Hierarchical Factor Model. Psychometrika.

[70] Reise S.P., Morizot J., Hays R.D. (2007). The role of the bifactor model in resolving dimensionality issues in health outcomes measures. Qual. Life Res..

[71] Chen F.F., West S., Sousa K. (2006). A Comparison of Bifactor and Second-Order Models of Quality of Life. Multivar. Behav Res..

[72] Beauducel A. (2011). Indeterminacy of Factor Score Estimates In Slightly Misspecified Confirmatory Factor Models. J. Mod. Appl. Stat. Methods.

[73] Johnson K. (2016). Realism and Uncertainty of Unobservable Common Causes in Factor Analysis. Noûs.

[74] Waller N.G. (2023). Breaking Our Silence on Factor Score Indeterminacy. J. Educ. Behav. Stat..

[75] Grice J.W. (2001). Computing and evaluating factor scores. Psychol. Methods.

[76] Lovie P., Lovie A.D. (1995). The cold equations: Spearman and Wilson on factor indeterminacy. Br. J. Math. Stat. Psychol..

[77] Logan J.A.R., Jiang H., Helsabeck N., Yeomans-Maldonado G. (2022). Should I allow my confirmatory factors to correlate during factor score extraction? Implications for the applied researcher. Qual. Quant..

[78] Kessy A., Lewin A., Strimmer K. (2018). Optimal Whitening and Decorrelation. Am. Stat..

[79] Strimmer K., Jendoubi T., Kessy A., Lewin A. (2021). Whitening: Whitening and High-Dimensional Canonical Correlation Analysis.

[80] Waller N.G., Jones J., Giordano C. (2022). fungible: Psychometric Functions from the Waller Lab.

[81] Rosseel Y. (2012). lavaan: An R Package for Structural Equation Modeling. J. Stat. Softw..

[82] Wang J., Qiu Y., Zhu X. (2023). Trends of mental health care utilization among US adults from 1999 to 2018. BMC Psychiatry.

[83] Widayana I.G., Agustina H., Mediawati A. (2025). Factors Associated with Work Life Balance Among Nurses in Hospitals: A Socio-Ecological Scoping Review. J. Multidiscip. Health.

[84] Micali E., Chiarella E.G. (2023). Burnout prevention in healthcare professionals during COVID-19. J. Prev. Med. Hyg..

[85] Cheng C.K.T., Chua J.H., Cheng L.J., Ang W.H.D., Lau Y. (2022). Global prevalence of resilience in health care professionals: A systematic review, meta-analysis and meta-regression. J. Nurs. Manag..

[86] Siswadi A.G.P., Shabrina A., Djunaidi A., Iskandarsyah A. (2022). The Role of Perceived Social Support and Resilience in Predicting the Mental Health of Healthcare Professionals During the COVID-19 Pandemic: A Study from Indonesia. Open Psychol. J..

[87] Lazarus R.S., Folkman S. (1984). Stress, Appraisal, and Coping.

[88] Peña-Mora M.J., Espinosa-Tigre R.M. (2025). Factores de riesgo ergonómico asociados a trastornos musculoesqueléticos en personal de enfermería, servicio de emergencia, Cuenca-Ecuador. MQRInvestigar.

