# Supplementary material for: Resilience, Quality of Life, and Minor Mental Disorders in Nursing Professionals: A Study in Challenging Work Environments
Source: Int J Environ Res Public Health. 2025 Aug 31;22(9):1375. doi: 10.3390/ijerph22091375 (PMC12469435; doi:10.3390/ijerph22091375)
Supplement: Supplementary file 1 [file ijerph-22-01375-s001.zip › ijerph-3801869-supplementary.pdf]

Supplementary File S1: Strengthening the Reporting of Observational Studies in Epidemiology

Item No	Recommendation	Section/Page in Manuscript	Manuscript Content (Page Numbers)
**Title and abstract**			
**1a**	Indicate the study's design with a commonly used term in the title or the abstract	Abstract	Page 2
**1b**	Provide in the abstract an informative and balanced summary of what was done and what was found	Abstract	Page 2
**Introduction**			
**2**	Explain the scientific background and rationale for the investigation being reported	Section 1. Introduction	Pages 2-5
**3**	State specific objectives, including any prespecified hypotheses	Section 1. Introduction	Page 5
**Methods**			
**4**	Present key elements of study design early in the paper	Section 2.1. Study Design	Page 5
**5**	Describe the setting, locations, and relevant dates, including periods of recruitment, exposure, follow-up, and data collection	Section 2.2. Sample and Context<br>Section 2.3. Data Collection	Page 6
**6a**	Give the eligibility criteria, and the sources and methods of selection of participants	Section 2.2. Sample and Context<br>Section 2.3. Data Collection	Page 6
**7**	Clearly define all outcomes, exposures, predictors, potential confounders, and effect modifiers. Give diagnostic criteria, if applicable	Section 2.4. Instruments<br>Section 3.2. Prevalence of Minor Mental Disorders (MMD)	Pages 7-8, Page 10
**8***	For each variable of interest, give sources of data and details of methods of assessment (measurement). Describe comparability of assessment methods if there is more than one group	Section 2.4. Instruments	Pages 7-8

**9**	Describe any efforts to address potential sources of bias	Section 2.3. Data Collection<br>Section 4.1. Limitations	Page 6, Pages 27-28
**10**	Explain how the study size was arrived at	Section 2.2. Sample and Context	Page 6
**11**	Explain how quantitative variables were handled in the analyses. If applicable, describe which groupings were chosen and why	Section 2.4. Instruments<br>Section 2.5. Data Analysis<br>Section 3.3.2. Wagnild and Young Resilience Scale	Pages 7-8, Page 15
**12a**	Describe all statistical methods, including those used to control for confounding	Section 2.5. Data Analysis<br>Section 2.5.1. Bayesian Confirmatory Factor Analysis (BCFA)<br>Section 2.5.2. Estimation and Transformation of Factor Scores<br>Section 2.5.3. Path Analysis Modeling	Pages 8-9
**12b**	Describe any methods used to examine subgroups and interactions	Section 3.5. Quality of Life and Minor Mental Disorders by Sociodemographic and Professional Data	Page 23, Pages 23-24 (Table 9), Pages 25-26 (Table 10)
**12c**	Explain how missing data were addressed	N/A	Information not explicitly detailed within pages 1-28.
**12d**	If applicable, describe analytical methods taking account of sampling strategy	Section 2.2. Sample and Context	Page 6
**12e**	Describe any sensitivity analyses	Section 2.5.3. Path Analysis Modeling	Page 9
**Results**			
**13a**	Report numbers of individuals at each stage of study—e.g., numbers potentially eligible, examined for eligibility, confirmed eligible, included in the study, completing follow-up, and analysed	Section 2.2. Sample and Context<br>Section 3.1. Sample Characterization	Page 6, Page 10

**13b**	Give reasons for non-participation at each stage	Section 2.3. Data Collection	Page 6
**13c**	Consider use of a flow diagram	N/A	Flow diagram not present.
**14a**	Give characteristics of study participants (e.g., demographic, clinical, social) and information on exposures and potential confounders	Section 3.1. Sample Characterization<br>Table 9<br>Table 10	Page 10, Pages 23-24, Pages 25-26
**14b**	Indicate number of participants with missing data for each variable of interest	N/A	Information not explicitly detailed within pages 1-28.
**15**	Report numbers of outcome events or summary measures	Section 3.2. Prevalence of Minor Mental Disorders (MMD)<br>Figure 12	Page 10
**16a**	Give unadjusted estimates and, if applicable, confounder-adjusted estimates and their precision (e.g., 95% confidence interval). Make clear which confounders were adjusted for and why they were included	Section 3.4. Path Analysis Results<br>Table 8	Page 19, Pages 19-22
**16b**	Report category boundaries when continuous variables were categorized	Section 2.4. Instruments<br>Section 3.3.2. Wagnild and Young Resilience Scale	Page 7, Page 15
**16c**	If relevant, consider translating estimates of relative risk into absolute risk for a meaningful time period	N/A	Not applicable for this cross-sectional study.
**17**	Report other analyses done—e.g., analyses of subgroups and interactions, and sensitivity analyses	Section 3.5. Quality of Life and Minor Mental Disorders by Sociodemographic and Professional Data<br>Section 2.5.3. Path Analysis Modeling	Page 23, Pages 23-24 (Table 9), Pages 25-26 (Table 10), Page 9
**Discussion**			

**18**	Summarise key results with reference to study objectives	Section 4. Discussion	Page 26
**19**	Discuss limitations of the study, taking into account sources of potential bias or imprecision. Discuss both direction and magnitude of any potential bias	Section 4.1. Limitations	Pages 27-28
**20**	Give a cautious overall interpretation of results considering objectives, limitations, multiplicity of analyses, results from similar studies, and other relevant evidence	Section 4. Discussion	Pages 26-27
**21**	Discuss the generalisability (external validity) of the study results	Section 3.1. Sample Characterization<br>Section 4. Discussion	Page 10, Page 27
**Other information**			
**22**	Give the source of funding and the role of the funders for the present study and, if applicable, for the original study on which the present article is based	Section 5. Funding	Information not found within pages 1-28. (Found on page 31)
